# Magnetization Transfer BOOST Noncontrast Angiography Improves Pulmonary Vein Imaging in Adults With Congenital Heart Disease

**DOI:** 10.1002/jmri.28280

**Published:** 2022-06-01

**Authors:** Imran Rashid, Giulia Ginami, Giovanna Nordio, Anastasia Fotaki, Radhouene Neji, Harith Alam, Kuberan Pushparajah, Alessandra Frigiola, Israel Valverde, René M. Botnar, Claudia Prieto

**Affiliations:** ^1^ School of Biomedical Engineering and Imaging Sciences King's College London London UK; ^2^ School of Medicine Case Western Reserve University Cleveland Ohio USA; ^3^ MR Research Collaborations Siemens Healthcare Limited Frimley UK; ^4^ Guy's and St Thomas' Hospital Department of Cardiology London UK; ^5^ Paediatric Cardiology Unit Hospital Virgen del Rocio and Institute of Biomedicine of Seville, IBIS Ciber‐CV Seville Spain

**Keywords:** magnetization Transfer, MTC‐BOOST, 3D whole heart imaging, congenital heart disease

## Abstract

**Background:**

Cardiac MRI plays an important role in the diagnosis and follow‐up of patients with congenital heart disease (CHD). Gadolinium‐based contrast agents are often needed to overcome flow‐related and off‐resonance artifacts that can impair the quality of conventional noncontrast 3D imaging. As serial imaging is often required in CHD, the development of robust noncontrast 3D MRI techniques is desirable.

**Purpose:**

To assess the clinical utility of noncontrast enhanced magnetization transfer and inversion recovery prepared 3D free‐breathing sequence (MTC‐BOOST) compared to conventional 3D whole heart imaging in patients with CHD.

**Study type:**

Prospective, image quality.

**Population:**

A total of 27 adult patients (44% female, mean age 30.9 ± 14.8 years) with CHD.

**Field Strength/Sequence:**

A 1.5 T; free‐breathing 3D MTC‐BOOST sequence.

**Assessment:**

MTC‐BOOST was compared to diaphragmatic navigator‐gated, noncontrast T2 prepared 3D whole‐heart imaging sequence (T2prep‐3DWH) for comparison of vessel dimensions, lumen‐to‐myocardium contrast ratio (CR), and image quality (vessel wall sharpness and presence and type of artifacts) assessed by two experienced cardiologists on a 5‐point scale.

**Statistical Tests:**

Mann–Whitney test, paired Wilcoxon signed‐rank test, Bland–Altman plots. *P* < 0.05 was considered statistically significant.

**Results:**

MTC‐BOOST significantly improved image quality and CR of the right‐sided pulmonary veins (PV): (CR: right upper PV 1.06 ± 0.50 vs. 0.58 ± 0.74; right lower PV 1.32 ± 0.38 vs. 0.81 ± 0.73) compared to conventional T2prep‐3DWH imaging where the PVs were not visualized in some cases due to off‐resonance effects. MTC‐BOOST demonstrated resistance to degradation of luminal signal (assessed by CR) secondary to accelerated or turbulent flow conditions. T2prep‐3DWH had higher image quality scores than MTC‐BOOST for the aorta and coronary arteries; however, great vessel dimensions derived from MTC‐BOOST showed excellent agreement with standard T2prep‐3DWH imaging.

**Data Conclusion:**

MTC‐BOOST allows for improved contrast‐free imaging of pulmonary veins and regions characterized by accelerated or turbulent blood flow compared to standard T2prep‐3DWH imaging, with excellent agreement of great vessel dimensions.

**Evidence Level:**

1

**Technical Efficacy:**

Stage 2

Congenital heart disease (CHD) affects approximately 8–10:1000 live births globally with approximately 90% of this group surviving into adulthood.^1^, ^2^ With the adult CHD population continuing to grow, life‐long follow‐up and surveillance are often required to assess for potential late‐stage complications. Due to the heterogeneous and complex anatomy of patients with CHD, cardiac MRI plays a fundamental role in diagnosis, preprocedural planning, and follow‐up.^3^, ^4^, ^5^

Whole heart imaging using ECG‐triggered noncontrast enhanced (non‐CE) T2 prepared (T2prep) balanced steady‐state free precession (bSSFP) (T2prep‐3DWH) is commonly employed for anatomical assessment in CHD.^6^ Limitations of this approach include heterogeneous blood pool contrast in regions of turbulent flow and suboptimal visualization of pulmonary and coronary venous vasculature, in part due to off‐resonance effects.^7^, ^8^ This is an important consideration for CHD patients where valvular and vessel abnormalities are prevalent.^1^ Contrast‐enhanced MR angiography (CE‐MRA) with intravenous gadolinium‐based contrast agents can be used to improve imaging of vascular structures affected by luminal signal loss.^3^, ^9^, ^10^, ^11^ However, CE‐MRA is performed without the use of cardiac gating, which can impede the accurate assessment of vessel dimensions due to the presence of motion‐related artifacts that are most evident at the aortic root and coronary arteries.^12^, ^13^ Furthermore, as administration of gadolinium‐based contrast agents has known risks, such as nephrogenic systemic fibrosis in patients with significant renal dysfunction, and undetermined risks related to potential gadolinium retention, noncontrast imaging approaches are desirable.^14^ Black‐blood cardiac MRI is commonly employed and allows for noncontrast visualization of myocardium and vessel wall; however, the conventional approach using double inversion recovery is limited to 2D acquisitions with nonisotropic resolution. A recent study demonstrated the feasibility of 3D black‐blood fast spin echo techniques for whole heart CHD imaging; however, this technique failed to consistently visualize the coronary arteries, representing an important limitation of this approach.^15^

This proof‐of‐concept study aims to investigate the potential clinical utility of a recently developed noncontrast magnetization transfer (MTC) and inversion recovery (IR) prepared sequence, “MTC‐BOOST,” that simultaneously generates co‐registered 3D bright‐ and black‐blood whole heart images, for anatomical assessment in patients with CHD.

## Materials and Methods

The study was approved by the National Research Ethics Service (1/11/12 and 15/NS/0030) and written informed consent was obtained from each participant according to institutional guidelines. Twenty‐seven adult patients (58% male, mean age 30.9 ± 14.8 years) with a spectrum of CHD who were scheduled for a clinically indicated cardiac MRI were recruited to undergo additional imaging with MTC‐BOOST from 2018 to 2019.

### MTC‐BOOST Sequence

**FIGURE 1:**
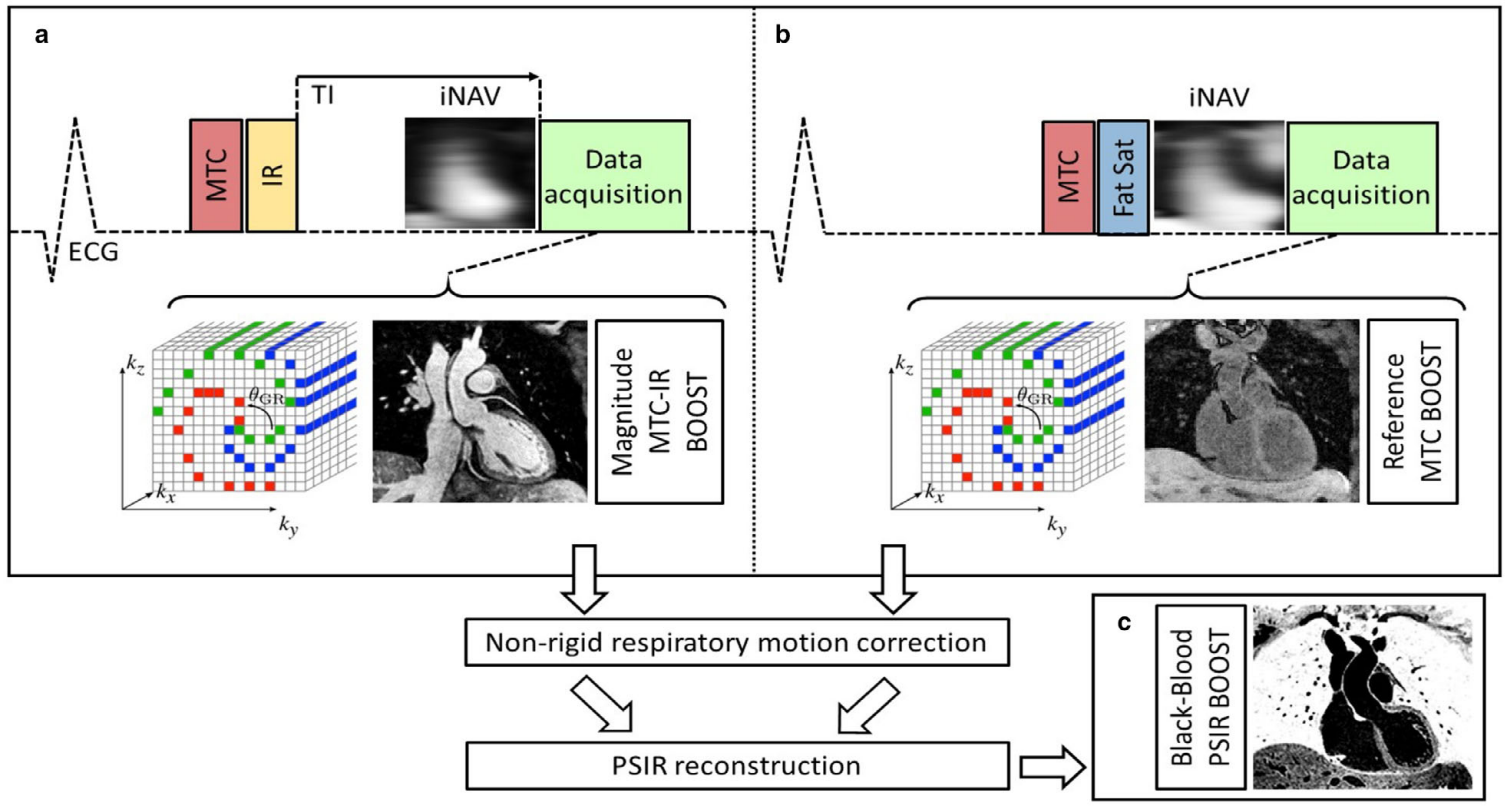
MTC‐BOOST pulse sequence diagram. Magnetization transfer (MTC) in combination with an inversion pulse (IR) is used in the odd heartbeats to acquire a magnitude volume (MTC‐IR BOOST, a), whereas only magnetization transfer preparation (MTC‐BOOST) is performed in the even heartbeats (b). Data acquisition is performed using a 3D Cartesian trajectory with spiral‐like order, segmented over multiple heartbeats. For respiratory motion correction, low‐resolution 2D image‐based navigator (iNAV) is acquired in each heartbeat. A nonrigid motion correction is applied on each volume, which are then combined with a PSIR reconstruction to generate a co‐registered black‐blood volume (c).

The data were acquired with an ECG‐triggered bSSFP 3D whole‐heart MTC‐BOOST imaging sequence,^16^ schematically shown in Fig. 1. MTC preparation in combination with an IR pulse is used in the odd heartbeats, whereas in the even heartbeats, only MTC preparation is performed. The bright blood MTC‐IR prepared acquisition (odd heartbeats) allows bright‐blood visualization of the heart anatomy and thoracic vascular structures including pulmonary arteries and veins. A phase sensitive inversion recovery (PSIR)‐like reconstruction enables the generation of a fully co‐registered 3D black blood dataset.^17^ In odd heartbeats, a short TI IR approach^18^ is used to suppress signal from epicardial fat (Fig. 1a), whereas frequency‐selective presaturation^19^ is used in even heartbeats for fat suppression (Fig. 1b). Data acquisition is performed using a 3D Cartesian trajectory with spiral‐like order^20^ and segmented over multiple heartbeats. For respiratory motion correction, low‐resolution 2D image navigators (iNAVs) are acquired in each heartbeat by spatially encoding ramp‐up pulses of the bSSFP sequences enabling beat‐to‐beat translational respiratory motion estimation and correction without data rejection (100% respiratory scan efficiency).^21^

### Motion Correction and Image Reconstruction

The MTC‐BOOST data were reconstructed offline using MATLAB (The MathWorks, Natick, MA). The MTC‐IR and MTC prepared volumes were independently reconstructed in end‐expiration using a previously described nonrigid motion correction framework.^22^ Briefly, right–left (RL) and superior–inferior (SI) displacements were estimated from the iNAVs, SI estimation was used to group the data in four to six equally populated bins, whereas both RL and SI estimations were used to correct for intrabin translational motion.^23^ Each bin was reconstructed with soft‐gated iterative SENSE. The bin corresponding to end‐expiration was then used as the reference bin to estimate bin‐to‐bin 3D nonrigid deformation fields via image registration.^24^ Successively the 3D nonrigid motion fields are incorporated in a generalized matrix description reconstruction pipeline where the motion‐corrected 3D volume is reconstructed using a linear conjugate gradient method.^25^ The motion‐corrected MTC‐IR and MTC prepared volumes were then combined in a PSIR‐like reconstruction to obtain a co‐registered black‐blood volume.

### Data Acquisition

Consecutive patients with CHD (*n* = 27) were scheduled for a clinically indicated cardiac MRI examination. Patients with intracardiac devices were excluded. Images were acquired on a 1.5 T MRI system (Magnetom Aera, Siemens Healthcare, Erlangen, Germany).

Data were acquired with an 18‐channel chest coil and a 32‐channel spine coil. The MTC‐BOOST imaging sequence was acquired precontrast administration with the following imaging parameters: coronal orientation, fully sampled with acquired spatial resolution of 1.4 × 1.4 × 2.8 mm, reconstructed isotropic resolution of 1.4 mm^3^, subject‐specific FOV = 320 × 320 × 90–120 mm^3^, TE = 1.5 msec, TR = 3.2 msec, flip angle for both odd and even heartbeats 90°, MT preparation: 15 Gaussian pulses, flip‐angle 800°, frequency offset 3000 Hz, duration 20.5 msec. In this study, a high‐frequency offset (3000 Hz) was chosen for MTC preparation to minimize image artifacts in regions with imperfect B_0_ and to minimize on‐resonance frequencies. Cine imaging was acquired in transverse orientation to determine the subject specific mid‐diastolic resting period (cardiac acquisition window ~120 msec, corresponding to 28–35 k‐space lines acquired per heartbeat). The established T2prep‐3DWH imaging was acquired using the following imaging parameters: spatial resolution 1.4 × 1.4 × 1.4 mm, T2prep of 40 msec, acceleration GRAPPA 2×, flip angle 90°. The T2prep‐3DWH sequence was combined with a conventional diaphragmatic (dNAV) navigator for respiratory motion compensation, with a gating window of ±3.5 mm in end‐expiration. Acquisition window was manually set to ~120 msec, depending on the subject‐specific mid‐diastolic resting period.

### Image Analysis

Image analysis was performed using Osirix v12.0 (Pixmeo, Geneva, Switzerland). The visualization of cardiac and vascular anatomy in CHD patients using bright‐blood MTC‐BOOST was compared to standard bright‐blood T2prep‐3DWH imaging. To this end, the lumen‐to‐myocardium contrast ratio (CR) was assessed for both the MTC‐BOOST bright blood and T2prep‐3DWH sequences.

CR measurements were taken in the following anatomical structures: right and left upper pulmonary vein (RUPV and LUPV), right and left lower pulmonary vein (RLPV and LLPV), left ventricle (LV), ascending aorta (Asc Ao), inferior vena cava (IVC), superior vena cava (SVC), right atrium (RA), right ventricle (RV), main pulmonary artery (MPA), left anterior descending coronary artery (LAD), left circumflex coronary artery (LCx) and the right coronary artery (RCA).

Image quality of the bright‐blood images of the MTC‐BOOST and T2prep‐3DWH imaging for each patient were assessed by two cardiologists (A.Fr. and I.V., Society for Cardiovascular Magnetic Resonance level III training). Each reader was blinded to the acquisition type they were grading and specific predefined anatomical structures were assessed: Asc Ao, MPA, right and left pulmonary veins (RPV and LPV), left main stem coronary artery (LM) and RCA. The image quality assessment was based on a 5‐point scoring system and was divided on sharpness of vessel borders (1 = nondiagnostic, 2 = poor, 3 = adequate, 4 = good, 5 = excellent) and presence of residual artifacts (1 = severe, 2 = moderate–severe, 3 = moderate, 4 = mild, and 5 = minimal or no artifact, where grades 1 and 2 represent nondiagnostic image quality). Overall diagnostic confidence of the MTC‐BOOST dataset was compared to the established T2prep‐3DWH. Diagnostic information was related to: 1) exclusion of anomalies, 2) suspected abnormalities, and 3) unsuspected abnormalities relevant to the patient's assessment regarding CHD. Level of confidence (1 = low, 2 = moderate, 3 = high, 4 = excellent).

Additionally, vessel dimensions were derived from both the bright‐blood and black‐blood MTC‐BOOST datasets and compared to the established T2prep‐3DWH. Vessel dimensions were measured using bright blood MTC‐BOOST, T2prep‐3DWH, and black blood MTC‐BOOST datasets at the level of the Asc Ao and proximal descending aorta (DAo), MPA, left (LPA) and right (RPA) branch pulmonary arteries. Blinded co‐axial measurements (max diameter) were performed using multiplanar reformats by a cardiologist (A.Fo, European Association of Cardiovascular Imaging level II training). For interobserver comparison, an additional cardiologist (I.R., Society for Cardiovascular Magnetic Resonance level III training) assessed aortic and pulmonary artery vessel dimensions derived from bright blood MTC‐BOOST imaging.

### Statistical Analysis

Statistical analysis was performed using IBM SPSS Statistics for Windows version 28.0.1.1 (IBM Corp, Armonk, N.Y., USA). Intraclass correlation coefficients and Bland–Altman plots were used to assess agreement of quantitative measurements of the previously defined anatomical structures using both MTC‐BOOST and T2prep‐3DWH imaging techniques. Image quality scores for vessel sharpness, presence of artifact and diagnostic confidence were assessed and compared using a paired Wilcoxon signed‐rank test. The total acquisition time of MTC‐BOOST and T2prep‐3DWH was compared using a Mann–Whitney test. Bland Altman plots were used to assess intersequence and interobserver agreement of vessel dimensions. A *P*‐value of <0.05 was considered statistically significant.

## Results

### Patient Characteristics and Cardiac MRI Study

**FIGURE 2:**
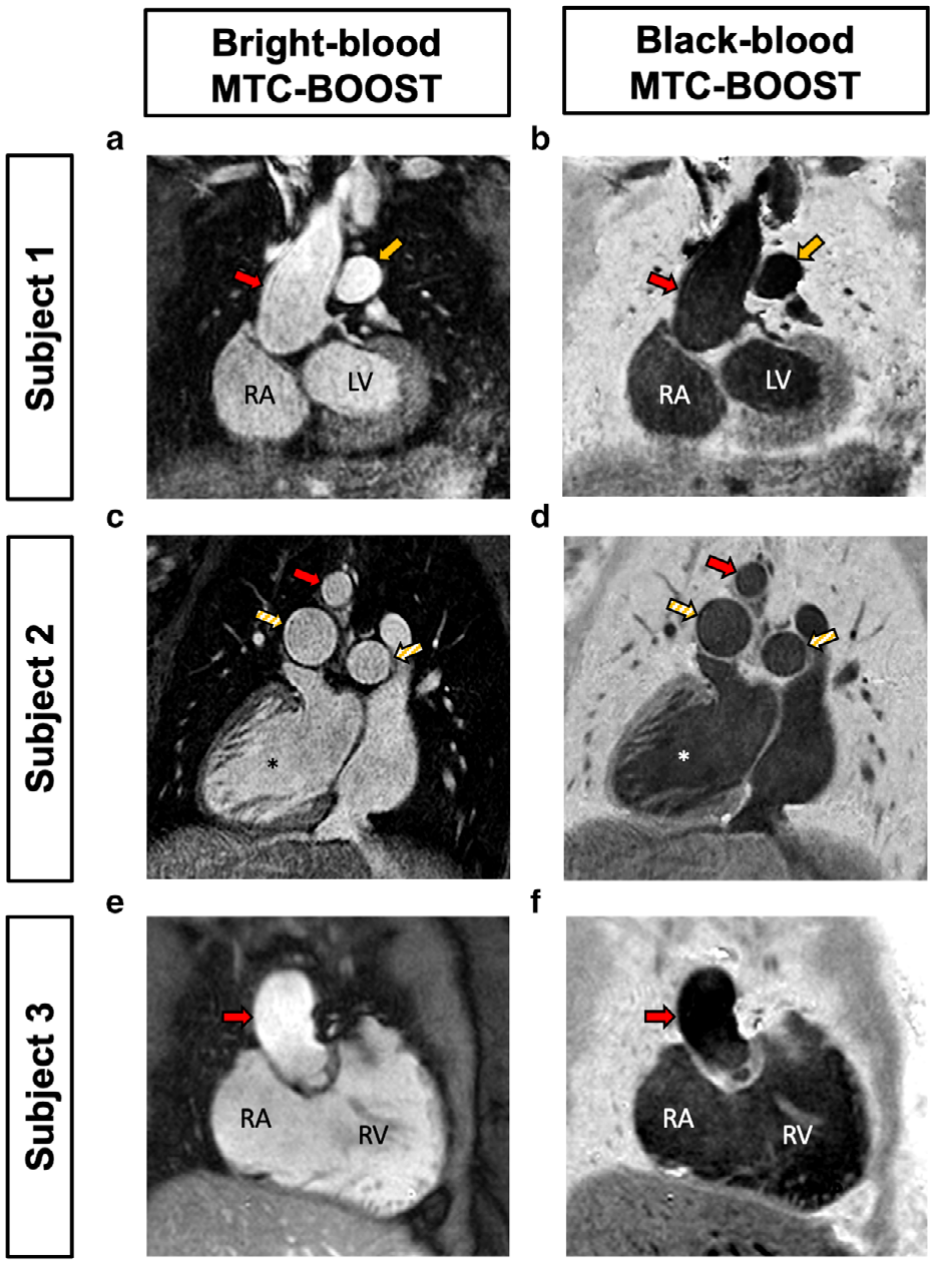
MTC‐BOOST derived bright‐ and black‐blood datasets in three representative patients with congenital heart disease. A single MTC‐BOOST acquisition yields co‐registered bright‐ and black‐blood 3D imaging datasets for: (a,b) prior subclavian flap repair of aortic coarctation (subject 1), (c,d) dextrocardia situs inversus (subject 2), (e,f) Tetralogy of Fallot with pulmonary valve replacement (subject 3). The bright blood MT‐IR prepared acquisition (odd heartbeats) allows bright‐blood visualization of the heart anatomy and thoracic vascular structures including pulmonary arteries and veins. A phase sensitive inversion recovery (PSIR)‐like reconstruction enables the generation of a black blood image from both odd and even heartbeats.

The patient characteristics are described in Table 1. Figure 2 shows the co‐registered bright‐ and black‐blood images acquired with the MTC‐BOOST imaging technique for three representative CHD patients with: subclavian flap repair of aortic coarctation (subject 1), dextrocardia situs inversus (subject 2) and repaired Tetralogy of Fallot (rTOF, subject 3), respectively. Supplementary Figure S1 depicts images from the MTC‐BOOST (bright and black blood datasets) vs. the conventional sequence in five representative views of the heart. The average scan time for MTC‐BOOST, that utilizes iNAV‐based respiratory motion compensation and generates both dark‐ and bright‐blood images, was lower than the standard T2prep‐3DWH that employs a diaphragmatic navigator to yield bright‐blood images only (9 ± 1.1 minutes vs. 11.1 ± 2.6 minutes). However, the slice thickness of MTC‐BOOST was double T2prep‐3DWH, and T2prep‐3DWH was undersampled (GRAPPA 2x) compared to fully sampled MTC‐BOOST, limiting direct comparisons of acquisition time.

**TABLE 1:** Patient Characteristics

Patient Characteristics	
Aortopulmonary window repair	1
Anomalous pulmonary venous drainage	4
Pulmonary stenosis, pulmonary valve atresia, tetralogy of fallot	5
Single ventricle Fontan	4
Aortic coarctation	5
Bicuspid aortic valve	5
PDA/ASD repair	1
Situs inversus dextrocardia	1
Left‐sided superior vena cava	1
Number of males (total number of patients)	15 (27)
Mean Age ± SD (year)	31 ± 15

PDA = patent ductus arteriosus; ASD = atrial septal defect; LSVC = left‐sided superior vena cava; SD = standard deviation.

### MTC‐BOOST Improved Pulmonary Vein Visualization Compared to T2prep‐3DWH Imaging

**FIGURE 3:**
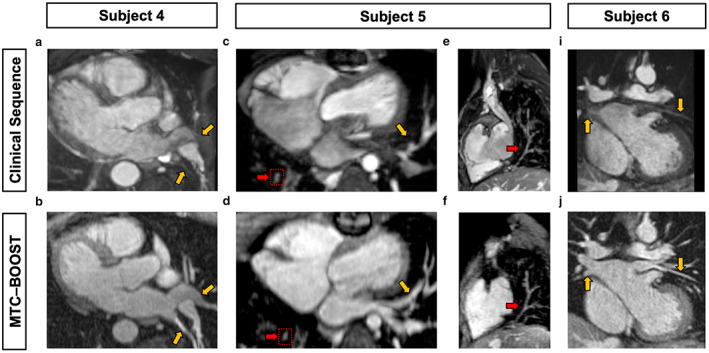
Comparison of MTC‐BOOST and standard T2prep 3D Whole Heart datasets in three representative patients for pulmonary vein imaging. Yellow arrows mark the sites of PVs draining into the LA. Subject 4 has surgically corrected partial anomalous pulmonary venous drainage (PAPVD), with poor delineation of the PVs using standard imaging (a) which are clearly visualized using MTC‐BOOST (b). Subject 5 has scimitar syndrome with an anomalous right pulmonary vein draining to the inferior vena cava. The left sided PVs (yellow arrows) are again poorly visualized with standard imaging (c) but clearly delineated with MTC‐BOOST (d). In the same patient, the anomalous right sided PV is clearly delineated in both sequences (red arrows, c–f). Subject 6 has PA hypoplasia, with luminal signal loss of both left and right sided PVs with the T2prep‐3DWH sequence (g) that is not apparent with MTC‐BOOST, where there is clear delineation of the PVs throughout their course (h). PV = pulmonary vein; PA = pulmonary artery; LA = left atrium.

MTC‐BOOST yielded marked improvements in pulmonary vein (PV) imaging compared to T2prep‐3DWH imaging sequences, in which luminal PV signal was reduced and, in some cases, could not be visualized. This was observed in a wide variety of anatomical variants with and without PV pathology (Fig. 3). This was clearly demonstrated in a patient with prior surgical correction of anomalous pulmonary venous drainage, where individual PVs were either not or only partially visualized adjacent to their left atrial insertion points using conventional T2prep‐3DWH imaging (Fig. 3a). In contrast, MTC‐BOOST in the same patient resulted in clear delineation of the PVs with uniform luminal signal (Fig. 3b). Similarly, in a patient with Scimitar syndrome, the left PV insertion points to the left atrium (LA) were not seen with standard T2prep‐3DWH imaging (Fig. 3c) but were clearly visualized in MTC‐BOOST (Fig. 3d). In the same patient, the anomalous right‐sided PV was detected in both T2prep‐3D whole heart and MTC‐BOOST acquisitions (Fig. 3e,f). Improved PV imaging with MTC‐BOOST was also clearly observed in a patient with pulmonary artery hypoplasia, where standard T2prep‐3DWH imaging showed bilateral PV luminal signal loss that was mitigated by MTC‐BOOST (Fig. 3g,h).

### MTC‐BOOST Conferred Resistance to Flow‐Induced Artifacts Compared to T2prep‐3DWH Imaging

**FIGURE 4:**
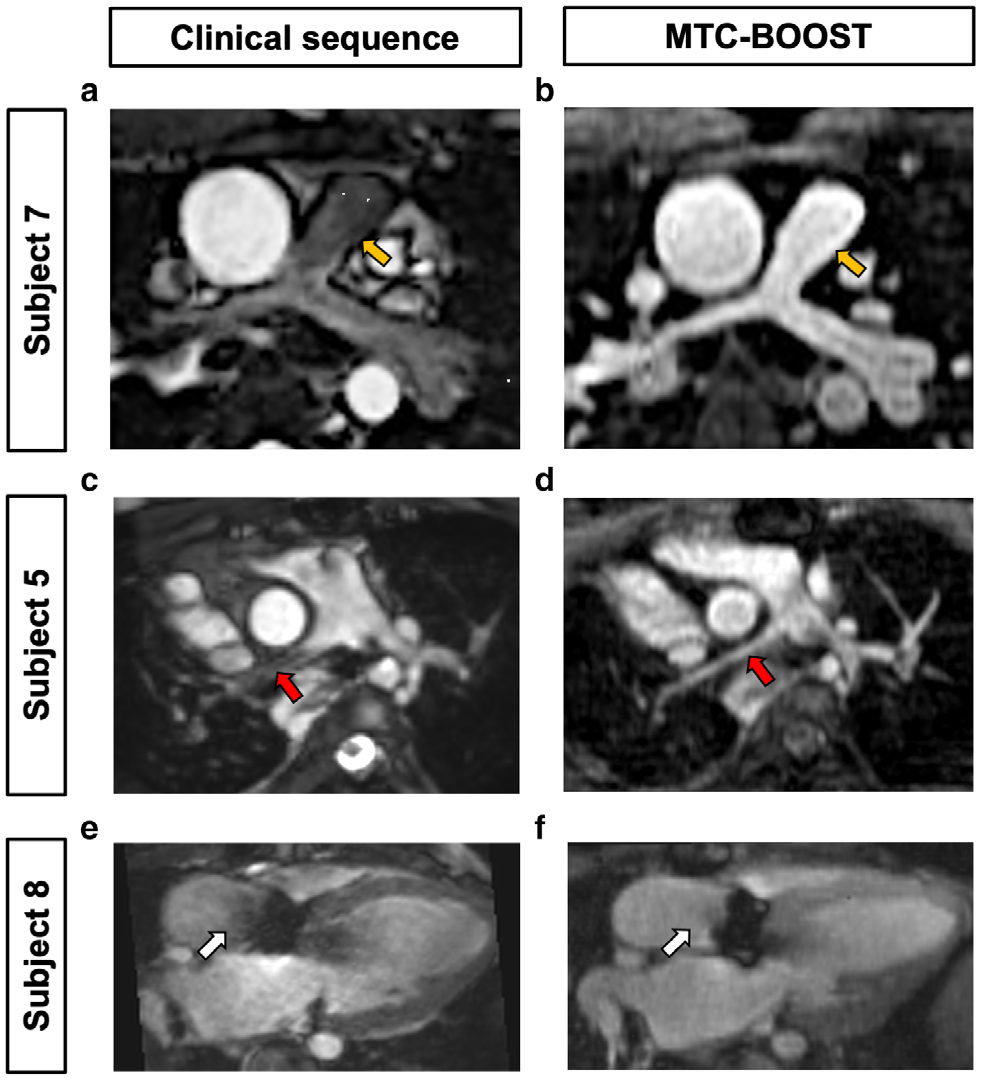
MTC‐BOOST shows resistance to flow‐mediated degradation of luminal signal in comparison to conventional T2prep‐3DWH imaging. Subject 7 has hypoplastic pulmonary arteries (PA) with reduced luminal signal of the main pulmonary artery (yellow arrows) and branch PA vessels using the standard T2prep‐3DWH sequence (a). In contrast, MTC‐BOOST results in clear delineation of the main PA (b). Subject 5 has scimitar syndrome with PA hypoplasia. There is reduced luminal signal in the right PA (red arrow, c) that is mitigated by MTC‐BOOST (d). Subject 8 has prosthetic aortic valve stenosis. There is nonuniform luminal signal in the proximal ascending aorta using the T2prep‐3DWH sequence (white arrow, e) which is not present in MTC‐BOOST images (f).

Many patients with CHD have vascular or valvular abnormalities that can result in accelerated or turbulent blood flow that often results in luminal signal loss on standard T2prep‐3DWH imaging due to dephasing of the MRI signal. Examples of such luminal signal loss were observed following T2prep‐3DWH imaging in patients with hypoplastic PAs associated with accelerated blood flow (Fig. 4a,c). In contrast, corresponding MTC‐BOOST images (Fig. 4b,d) yielded a higher luminal CR compared to T2prep‐3DWH (CR: 1.3 vs. 0.8, and 1.9 vs. 0.8 for subjects 5 and 7, respectively). MTC‐BOOST also provided a reduction in luminal signal loss compared to T2prep‐3DWH due to turbulent flow from stenotic valves as demonstrated in a patient with recurrent aortic stenosis following aortic valve replacement (CR: 1.4 vs. 0.9; Fig. 4e,f).

### Caval Imaging in Fontan Patients Using MTC‐BOOST

**FIGURE 5:**
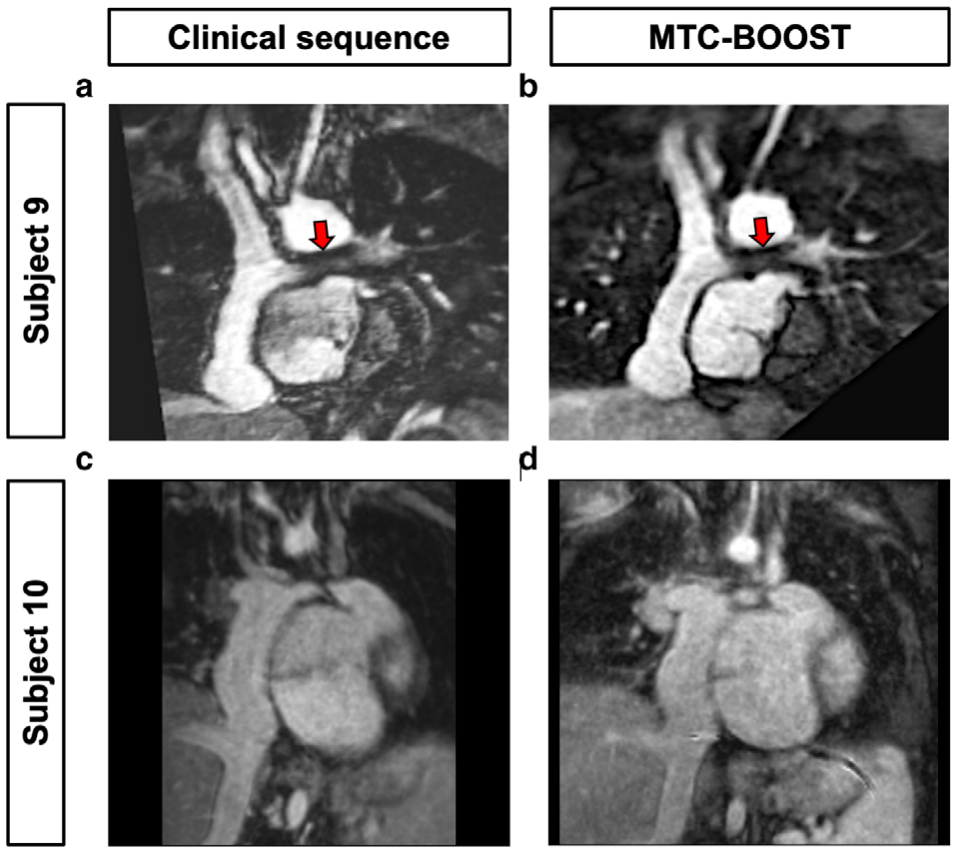
MTC‐BOOST in single ventricle Fontan patients. Both MTC‐BOOST (b, d) and standard T2 prepared whole heart sequences (a, c) produced clear delineation of the Fontan pathways in patients with a single ventricle and prior Fontan palliation. Subject 9 has a prior left PA stent (red arrow), which caused similar loss of luminal signal on both MTC‐BOOST and T2prep‐3DWH sequences (a,b).

Comparison of MTC‐BOOST and conventional T2prep‐3DWH imaging was also performed in single ventricle Fontan patients (Fig. 5). Here, MTC‐BOOST showed good visualization of the superior and inferior vena cava, which are characterized by low‐velocity venous blood flow, to the branch PAs in patients who have undergone Fontan palliation (Fig. 5b,d). The luminal caval signal was similar to the standard T2prep‐3DWH imaging (Fig. 5a,c) performed in the same patients.

### Visualization of Stents

Neither bright‐ nor black‐blood MTC‐BOOST offers an advantage in comparison to the established T2prep‐3DWH imaging for artifact‐free intraluminal stent visualization. Artifact from a branch PA stent had a similar appearance in both MTC‐BOOST and T2prep‐3DWH acquisitions (CR: 0.53 vs. 0.58, respectively), (Fig. 5 red arrow, Supplementary Fig. S2).

### Assessment of Vessel Dimensions by MTC‐BOOST Compared to T2prep‐3DWH

**FIGURE 6:**
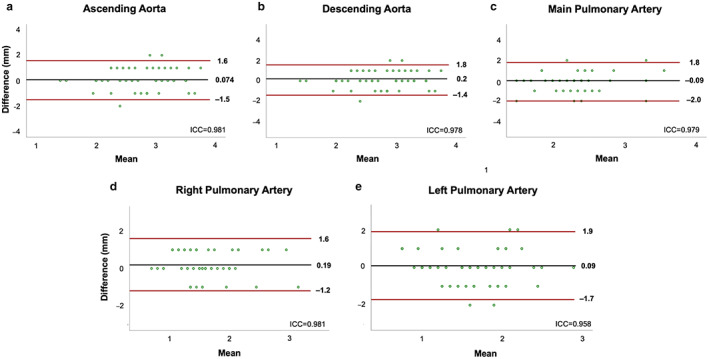
Bland–Altman plots for vessel dimension assessment comparing MTC‐BOOST and T2prep‐3DWH imaging sequences. Bland–Altman assessment of maximal co‐axial vessel dimensions of (a) ascending aorta at the level of the RPA, (b) descending thoracic aorta at the level of the RPA, (c) main pulmonary artery, (d) right pulmonary artery and (e) left pulmonary artery derived from multiplanar reformats of bright‐blood MTC‐BOOST and standard T2prep‐3DWH datasets. Black lines denote mean difference and red lines denote 95% CI (±1.96 SD). Intraclass correlation coefficients (ICC) between T2prep‐3DWH and MTC‐BOOST are included. RPA = right pulmonary artery; MTC = magnetization transfer contrast.

Aortic dimensions demonstrate excellent agreement between bright blood MTC‐BOOST and T2prep‐3DWH sequences for the assessment of vessel dimensions at different anatomical regions (Fig. 6) including the mid ascending aorta (mean difference: 0.074 mm, 95% confidence interval [CI]: −1.5 to 1.6), the descending thoracic aorta (mean difference: 0.2 mm, 95% CI:−1.4 to 1.8), the main pulmonary artery (mean difference: −0.09 mm, 95% CI: −2.0 to 1.8) and branch PAs. Interobserver comparison of great vessel dimensions (A.Fo. and I.R.) showed excellent agreement between readers for the ascending aorta (mean difference: 0.1 mm, 95% CI: −1.8 to 2) and main pulmonary artery (mean difference: −0.2 mm, 95% CI: −2.2 to 1.8) (Supplementary Fig. S3). Additionally, comparison of vessel dimensions derived from black blood MTC‐BOOST imaging and the T2prep‐3DWH using Bland–Altman analysis demonstrated excellent agreement between both sequences across all measured vessel diameters (Supplementary Fig. S4).

### Luminal to Myocardial CR of MTC‐BOOST Compared to Standard T2prep‐3DWH Imaging

The lumen‐to‐myocardium CR results of the great vessels and cardiac chambers demonstrate significantly higher right upper (1.06 ± 0.50 vs. 0.58 ± 0.74) and right lower (1.32 ± 0.38 vs. 0.81 ± 0.73) pulmonary vein contrast ratios for MTC‐BOOST compared to T2prep‐3DWH imaging, respectively. The T2prep‐3DWH sequence yielded lumen to myocardium contrast ratios lower than 1, consistent with a marked degradation of luminal signal (Supplementary Fig. S5). There was no significant difference in the CR of the left upper and left lower PV between the two sequences (*P* = 0.09 and *P* = 0.08, respectively). The contrast ratios for the aorta, inferior and superior vena cava, cardiac chambers and left anterior descending coronary artery were higher using the T2prep‐3DWH sequence.

### MTC‐BOOST Image Quality Assessment

MTC‐BOOST resulted in significantly superior image quality compared to T2prep‐3DWH with regard to both vessel sharpness (Table 2) and the presence of artifact (Table 3) for the right pulmonary veins (vessel sharpness, mean ± SD, reviewer 1: 4 ± 0.6 vs. 3.3 ± 0.8, reviewer 2: 4.5 ± 0.9 vs. 1.4 ± 0.7; presence of artifact, reviewer 1: 4 ± 0.7 vs. 3.3 ± 0.8, reviewer 2: 3.3 ± 0.8 vs. 1.6 ± 0.6) and left pulmonary veins (vessel sharpness, reviewer 1: 4.1 ± 0.6 vs. 3.1 ± 0.6, reviewer 2: 4.4 ± 1 vs. 1.7 ± 0.8; presence of artifact, reviewer 1: 4.1 ± 0.6 vs. 3.1 ± 0.6, reviewer 2: 4 ± 1 vs. 1.7 ± 0.7). There was no significant difference in vessel sharpness (*P* = 0.6 and 0.5) or presence of artifact (*P* = 0.9 and 0.7) as assessed by image quality scores for the main pulmonary artery for either reader (Tables 2 and 3). Image quality scores for the coronary arteries were higher using the conventional T2prep‐3DWH imaging compared to MTC‐BOOST (Tables 2 and 3); however, mean quality scores for MTC‐BOOST ranged from 2.7 to 3.2 for vessel sharpness and 2.7 to 4 for the presence of artifact, indicating that the majority of MTC‐BOOST acquisitions achieved diagnostic image quality. The diagnostic confidence following analysis of MTC‐BOOST bright‐ and black‐blood datasets was compared to T2prep‐3DWH and showed significant superiority of the proposed sequence with a mean quality score of 3.7 ± 0.5 for both the bright‐ and black‐blood datasets versus 3.2 ± 0.8 for the T2prep‐3DWH sequence. In 7 out of the 27 datasets, the T2prep‐3DWH sequence had a lower quality score because of poorer delineation of the pulmonary veins and in six additional datasets, this was combined with artifacts in the pulmonary arteries. In three datasets, the bright‐ and black‐blood MTC‐BOOST sequences had a lower score due to poorer delineation of the coronary arteries.

**TABLE 2:** Image Quality Scores—Vessel Sharpness of the Coronary Arteries and Great Vessels

Vessel Sharpness	Clinical (T2 prepared 3D whole heart)	MTC‐BOOST	*P* value
LM (R1)	3.9 ± 1.0	3.2 ± 0.7	0.07
LM (R2)	3.9 ± 1.5	3.0 ± 1.6	<0.05
RCA (R1)	3.7 ± 1.3	2.7 ± 1.1	<0.05
RCA (R2)	3.8 ± 1.4	2.8 ± 1.4	<0.05
Aorta (R1)	4.6 ± 0.6	4.2 ± 0.7	<0.05
Aorta (R2)	4.6 ± 0.8	4.3 ± 0.9	0.1
MPA (R1)	4.3 ± 0.9	4.2 ± 0.8	0.6
MPA (R2)	3.8 ± 1.4	4.0 ± 1.1	0.5
RPV (R1)	3.3 ± 0.8	4.0 ± 0.6	<0.05
RPV (R2)	1.4 ± 0.7	4.5 ± 0.9	<0.05
LPV (R1)	3.1 ± 0.6	4.1 ± 0.6	<0.05
LPV (R2)	1.7 ± 0.8	4.4 ± 1.0	<0.05

Image quality scores—vessel wall sharpness. LM = left main coronary artery; RCA = right coronary artery; MPA = main pulmonary artery; RPV = right‐sided pulmonary veins; LPV = left‐sided pulmonary veins. Two blinded reviewers (R1, R2) assessed the clinical sequence (T2 prepared 3D whole heart) and MTC‐BOOST bright blood datasets to grade the sharpness of vessel borders (1 = nondiagnostic, 2 = poor, 3 = adequate, 4 = good, 5 = excellent). For each observer, the scores were compared using a paired Wilcoxon signed‐rank test, with statistical significance difference set to *P* < 0.05.

**TABLE 3:** Image Quality Scores—Coronary Artery and Great Vessel Artifact

Presence of Artifact	Clinical	MTC‐BOOST	*P* value
LM (R1)	4.1 ± 1.0	3.3 ± 0.7	<0.05
LM (R2)	3.7 ± 1.5	4.0 ± 1.0	0.5
RCA (R1)	4 ± 1.2	2.9 ± 1.1	<0.05
RCA (R2)	3.9 ± 1.4	2.7 ± 1.3	<0.05
Aorta (R1)	4.7 ± 0.6	4.2 ± 0.5	<0.05
Aorta (R2)	4.3 ± 1.0	3.7 ± 0.7	<0.05
MPA (R1)	4.2 ± 0.9	4.2 ± 0.8	0.9
MPA (R2)	3.8 ± −1.4	3.8 ± 0.8	0.7
RPV (R1)	3.3 ± 0.8	4.0 ± 0.7	<0.05
RPV (R2)	1.6 ± 0.6	3.3 ± 0.8	<0.05
LPV (R1)	3.1 ± 0.6	4.1 ± 0.6	<0.05
LPV (R2)	1.7 ± 0.7	4.0 ± 1.0	<0.05

Image quality scores—presence of artifact. LM = left main coronary artery; RCA = right coronary artery; MPA = main pulmonary artery; RPV = right‐sided pulmonary veins; LPV = left‐sided pulmonary veins. Two blinded reviewers (R1, R2) assessed the clinical sequence (T2 prepared 3D whole heart) and MTC‐BOOST bright blood datasets to grade presence of artifact (1 = severe artifact, 2 = significant artifact, 3 = moderate artifact, 4 = mild artifact, and 5 = minimal artifact) where grades 1 and 2 delineate nondiagnostic datasets. For each observer, the scores were compared using a paired Wilcoxon signed‐rank test, with statistical significance difference set to *P* < 0.05.

## Discussion

In this study, we demonstrated the feasibility of free‐breathing 3D whole heart MT‐IR prepared BOOST (MTC‐BOOST) for contrast‐free imaging of CHD patients. MTC‐BOOST simultaneously yields a bright‐blood and a complementary co‐registered black‐blood 3D whole‐heart volume. This proof‐of‐concept study compared MTC‐BOOST to the established bright‐blood T2prep‐3DWH imaging technique for the visualization of cardiac and vascular anatomy in CHD patients, including patients with obstructive right and left heart disease, single ventricle pathology and anomalous pulmonary venous drainage. This broad case mix allowed for an assessment of the performance of MTC‐BOOST compared to the conventional T2prep‐3DWH imaging sequence in an anatomically diverse population. The results demonstrate excellent agreement between the two sequences in terms of vessel dimensions with improved visualization in MTC‐BOOST of the pulmonary venous vasculature and regions of accelerated or turbulent flow, which is likely conferred by resistance to off‐resonance artifacts. In addition, a wide spectrum of CHD patients was represented in the current study, including complex CHD, single ventricle palliation, pulmonary venous abnormalities, valvular and great artery disease, supporting the applicability of MTC‐BOOST to the broader CHD population.

Conventional T2prep‐3DWH sequences often demonstrated severe artifacts in the PV and occasionally LA. The reduction in luminal signal observed in PVs with conventional T2prep‐3DWH imaging is likely mediated by off‐resonance effects as previously described by Hu et al, who demonstrated that PV blood off‐resonance effects were responsible for signal void artifacts in the PV vasculature when imaged with 3D noncontrast enhanced T2 prepared SSFP sequences.^7^ The same study also demonstrated that PV blood flow, in addition to off‐resonance, can cause signal void artifacts in the LA. In contrast, the MT‐IR bright blood component of the MTC‐BOOST sequence yielded clear and consistent delineation of PVs associated with significantly higher image quality scores for the right PVs with a corresponding increase in luminal‐to‐myocardial CR. The contrast‐to‐noise ratio was not used for comparison due to different reconstruction methods employed leading to significant variation in background noise between datasets. The exact cause of the higher susceptibility of the right‐sided pulmonary veins to off‐resonance and luminal signal reduction using conventional T2prep‐3DWH sequences remains unclear. It may be related to their proximity to the lung parenchyma or to the use of pencil‐beam navigators placed over the right hemidiaphragm. Furthermore, the higher and more uniform luminal signal in regions of accelerated or turbulent blood flow observed with MTC‐BOOST support the resistance of MT preparation to flow‐mediated off‐resonance effects compared to standard T2 prepared acquisitions. For other structures including the aorta, inferior and superior vena cava, cardiac chambers and left anterior descending coronary artery, T2prep‐3DWH yielded higher lumen to myocardium contrast ratios. However, these structures were clearly visualized using both sequences and the observed differences in CR for the great vessels did not significantly impact image quality.

The improved noncontrast imaging of regions of accelerated or turbulent blood flow and the pulmonary venous vasculature observed with MTC‐BOOST has multiple potential applications in CHD patients. CHD is characterized by regions of turbulent blood flow (eg, hypoplastic vessels or coarctation, vessel anastomoses, and stenotic or regurgitant valves) and MTC‐BOOST may allow for a detailed anatomical assessment in these regions without the need for contrast administration. Furthermore, improved imaging of the pulmonary venous vasculature has several potential advantages in CHD, including patients with Fontan circuits, where elevated central venous pressures can promote the development of venovenous collateral vessels. As the pulmonary veins are a common site of terminal drainage of venous collateral vessels with potential for right‐to‐left shunt, clear imaging of the pulmonary vasculature is needed to accurately identify these shunts, which can be a target for intervention. The clear delineation of the pulmonary veins also allows for the assessment of partial anomalous pulmonary venous drainage (PAPVD) and pulmonary venous stenosis, which is limited using standard noncontrast‐enhanced T2prep‐3DWH sequences. The identification of left‐to‐right shunt on flow quantification studies often necessitates exclusion of anomalous pulmonary venous drainage and the proposed sequence is able to achieve this task without the administration of a contrast agent. The reduction in gadolinium utilization could lower costs and the risk of contrast‐related complications. Additionally, the higher diagnostic confidence associated with MTC‐BOOST may reduce the need for additional imaging techniques such as CT.

The MTC‐BOOST framework, as previously demonstrated,^16^ was integrated with image‐based navigation. This allowed for data acquisition with nearly 100% respiratory scan efficiency and with predictable acquisition time. The use of a diaphragmatic navigator (dNAV) gating to account for respiratory motion introduces problems, such as prolonged and unpredictable scan times due to the fact that data acquired outside a pre‐defined respiratory window (usually end‐expiration) is rejected and needs to be reacquired. Furthermore, dNAV provides only an indirect estimation of the respiratory motion of the heart, as it tracks the SI displacement of the dome of the right hemidiaphragm. In addition, the intersection between the dNAV and the PVs may cause inflow artifacts and lead to reduced sharpness of such vessels.^26^, ^27^ MTC‐BOOST relies on the acquisition of two differently weighted bright‐blood volumes (over two heart beats) for two reasons. First, to allow recovery from the IR prepulse and improve visualization of the vessels in the bright‐blood volume and second, to provide a complementary co‐registered black‐blood PSIR volume without time penalty. This study has demonstrated excellent agreement in vascular and cardiac measurements derived from both the bright‐ and black‐blood MTC‐BOOST datasets with T2prep‐3DWH based dimensions. No particular advantage was observed when measuring vessel dimensions using black‐blood MTC‐BOOST images compared to the bright‐blood dataset. Unlike 2D spin echo black‐blood imaging, the 3D black‐blood MTC‐BOOST dataset does not allow for in‐stent luminal assessment.^28^ However, there is supporting evidence that PSIR carries important diagnostic information for thrombus visualization that is relevant for Fontan and baffle (eg Mustard procedure) assessment.^29^ Future studies should further investigate the clinical value of the black‐blood MTC‐BOOST volume in the CHD population in comparison to conventional 2D spin echo black‐blood and recently proposed 3D black‐blood approaches.^29^

### Limitations

First, we compared noncontrast‐enhanced MTC‐BOOST against standard noncontrast T2prep‐3DWH but not against CE‐MRA. Future studies comparing MTC‐BOOST and CE‐MRA are required to assess whether the need for contrast administration can be avoided. The number of subjects in the current study was limited and a larger study would allow for inclusion of a broader representation of CHD patients. Finally, MTC‐BOOST was acquired using anisotropic resolution, which is suboptimal for 3D multiplanar reformat assessment and may explain the lower image quality scores for coronary imaging with MTC‐BOOST in comparison with conventional (isotropic) T2 prepared 3D imaging. Forthcoming studies might benefit from an isotropic acquisition with higher spatial resolution combined with image acceleration techniques.

### Conclusion

MTC‐BOOST allows for single acquisition noncontrast assessment of cardiac, arterial, and venous anatomy in adult patients with CHD. It provides clear delineation of the pulmonary veins and regions of accelerated blood flow, which was not possible using conventional T2prep‐3DWH imaging techniques that are susceptible to signal voids due to turbulent flow and off‐resonance effects. MTC‐BOOST had lower image quality scores for the coronary arteries and aorta but yielded excellent agreement for great vessel dimensions. For the same spatial resolution, scan times for fully sampled MTC‐BOOST may be longer than T2prep3DWH (undersampled by factor of 2) and assessment of intravascular stent patency was not possible with either of these noncontrast sequences. Overall, as MTC‐BOOST improves visualization of the pulmonary veins and provides accurate great vessel dimensions compared to established clinical sequences, it holds promise for the assessment of CHD.

## Supporting information


**Supplementary Figure 1 MTC‐BOOST compared to T2prep3DWH in a patient with partial anomalous pulmonary venous drainage.** Bright‐ and black‐blood MTC‐BOOST datasets provide better delineation of the course of the anomalous left upper pulmonary vein (LUPV) to the superior vena cava (SVC), in comparison to the clinical sequence. MTC‐BOOST bright‐ and black‐blood have uniform signal within all cardiac structures demonstrated: left and right lower pulmonary veins (LPV), branch pulmonary arteries (BPA), left ventricular outflow tract (LVOT) and right ventricular outflow tract (RVOT). Red arrows denote the stated anatomical structure for each column.
**Supplementary Figure 2. Bright‐ and black‐blood MTC‐BOOST and T2prep3DWH imaging of Fontan circulation with LPA stent in situ.** Coronal and transverse imaging planes are shown. The LPA stent (red arrows) is associated with luminal signal reduction/void in T2prep‐3DWH, bright‐ and black‐blood MTC‐BOOST datasets.
**Supplementary Figure 3: Bland Altman analysis of vessel dimensions derived from MTC‐BOOST by different readers.** Blinded reader assessment of maximal luminal dimension (cm) of A) ascending aorta at the level of the RPA, and B) main pulmonary artery from multiplanar reformats of bright blood MTC‐BOOST by two different readers (A.Fo and I.R). Black lines denote mean difference, red lines denote 95% CI (±1.96SD).
**Supplementary Figure 4: Bland Altman analysis of vessel dimensions using black blood MTC‐BOOST and T2prep‐3DWH imaging.** Blinded reader assessment of maximal luminal dimension of A) ascending aorta at the level of the RPA, B) descending thoracic aorta at the level of the RPA, C) main pulmonary artery, D) right pulmonary artery and E) left pulmonary artery from multiplanar reformats of black blood MTC‐BOOST and T2prep‐3DWH imaging. Black lines denote mean difference, red lines denote 95% CI (±1.96SD).
**Supplementary Figure 5: Comparison of contrast ratio (lumen/myocardium) of the great vessels and cardiac chambers using MTC‐BOOST and standard clinical T2prep‐3DWH imaging.** Contrast ratios of the right upper (ruPV) and right lower (rlPV) pulmonary veins were significantly higher for the right‐sided pulmonary veins but not significantly different for the left upper (luPV) and left lower (llPV) pulmonary veins. Contrast ratios for the left ventricle (LV), ascending aorta (Asc Ao), inferior vena cava (IVC), superior vena cava (SVC), right atrium (RA), right ventricle (RV) and main pulmonary artery (MPA) were significantly higher for standard T2prep‐3DWH imaging compared to MTC‐BOOST, where all mean contrast ratios were greater than 1. *p < 0.05 by Mann–Whitney.
