# Supplementary material for: Magnetization Transfer BOOST Noncontrast Angiography Improves Pulmonary Vein Imaging in Adults With Congenital Heart Disease
Source: J Magn Reson Imaging. 2022 Jun 1;57(2):521–31. doi: 10.1002/jmri.28280 (PMC10084321; doi:10.1002/jmri.28280)
Supplement: Supplementary file 1 — Supplementary Figure 1 MTC‐BOOST compared to T2prep3DWH in a patient with partial anomalous pulmonary venous drainage. Bright‐ and black‐blood MTC‐BOOST datasets provide better delineation of the course of the anomalous left upper pulmonary vein (LUPV) to the superior vena cava (SVC), in comparison to the clinical sequence. MTC‐BOOST bright‐ and black‐blood have uniform signal within all cardiac structures demonstrated: left and right lower pulmonary veins (LPV), branch pulmonary arteries (BPA), left ventricular outflow tract (LVOT) and right ventricular outflow tract (RVOT). Red arrows denote the stated anatomical structure for each column. Supplementary Figure 2. Bright‐ and black‐blood MTC‐BOOST and T2prep3DWH imaging of Fontan circulation with LPA stent in situ. Coronal and transverse imaging planes are shown. The LPA stent (red arrows) is associated with luminal signal reduction/void in T2prep‐3DWH, bright‐ and black‐blood MTC‐BOOST datasets. Supplementary Figure 3: Bland Altman analysis of vessel dimensions derived from MTC‐BOOST by different readers. Blinded reader assessment of maximal luminal dimension (cm) of A) ascending aorta at the level of the RPA, and B) main pulmonary artery from multiplanar reformats of bright blood MTC‐BOOST by two different readers (A.Fo and I.R). Black lines denote mean difference, red lines denote 95% CI (±1.96SD). Supplementary Figure 4: Bland Altman analysis of vessel dimensions using black blood MTC‐BOOST and T2prep‐3DWH imaging. Blinded reader assessment of maximal luminal dimension of A) ascending aorta at the level of the RPA, B) descending thoracic aorta at the level of the RPA, C) main pulmonary artery, D) right pulmonary artery and E) left pulmonary artery from multiplanar reformats of black blood MTC‐BOOST and T2prep‐3DWH imaging. Black lines denote mean difference, red lines denote 95% CI (±1.96SD). Supplementary Figure 5: Comparison of contrast ratio (lumen/myocardium) of the great vessels and cardiac chambers using [file JMRI-57-521-s001.docx]

**Supplementary Material**

**
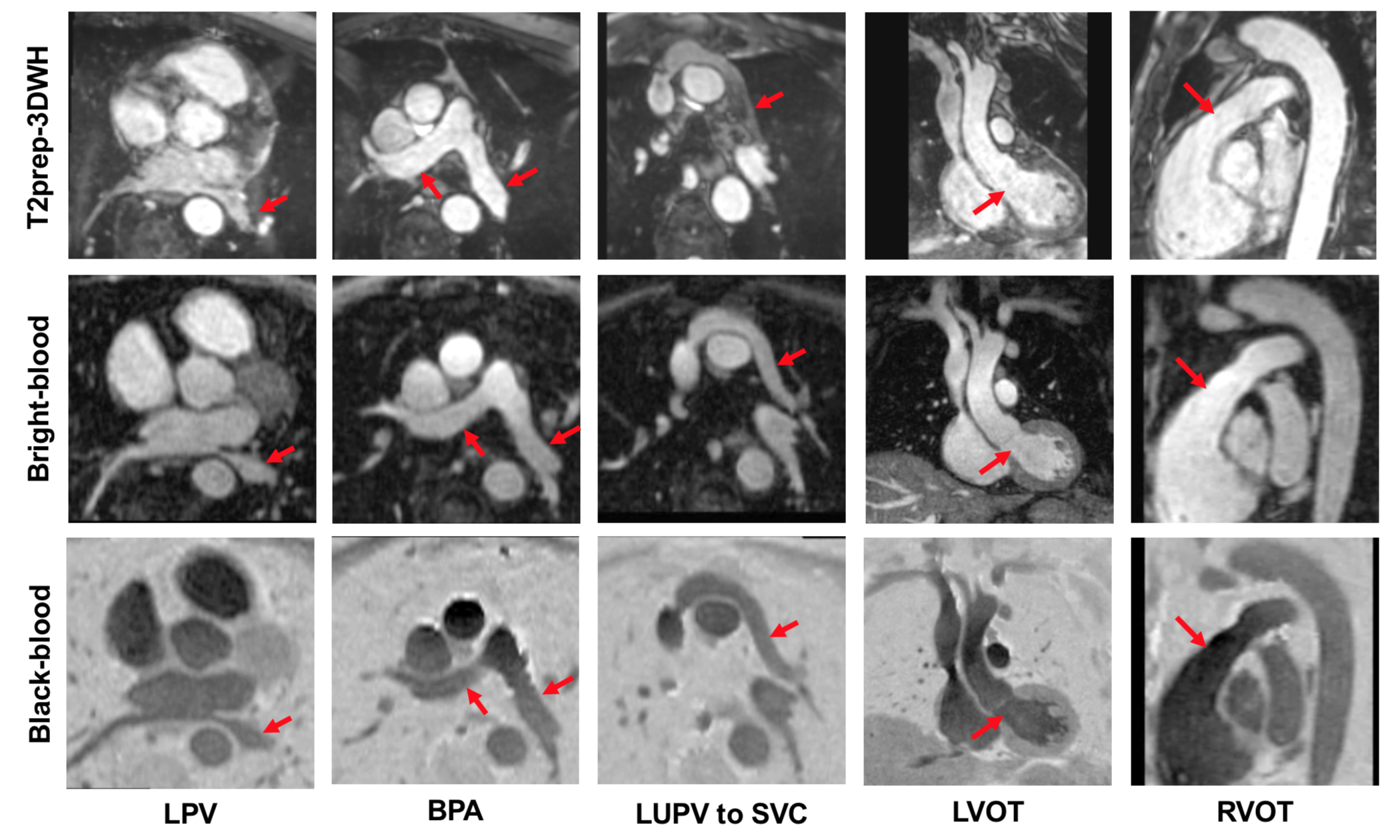
**

**Supplementary Figure 1. MTC-BOOST compared to T2prep3DWH in a patient with partial anomalous pulmonary venous drainage.** Bright- and black-blood MTC-BOOST datasets provide better delineation of the course of the anomalous left upper pulmonary vein (LUPV) to the superior vena cava (SVC), in comparison to the clinical sequence. MTC-BOOST bright- and black-blood have uniform signal within all cardiac structures demonstrated: left and right lower pulmonary veins (LPV), branch pulmonary arteries (BPA), left ventricular outflow tract (LVOT) and right ventricular outflow tract (RVOT). Red arrows denote the stated anatomical structure for each column.

**
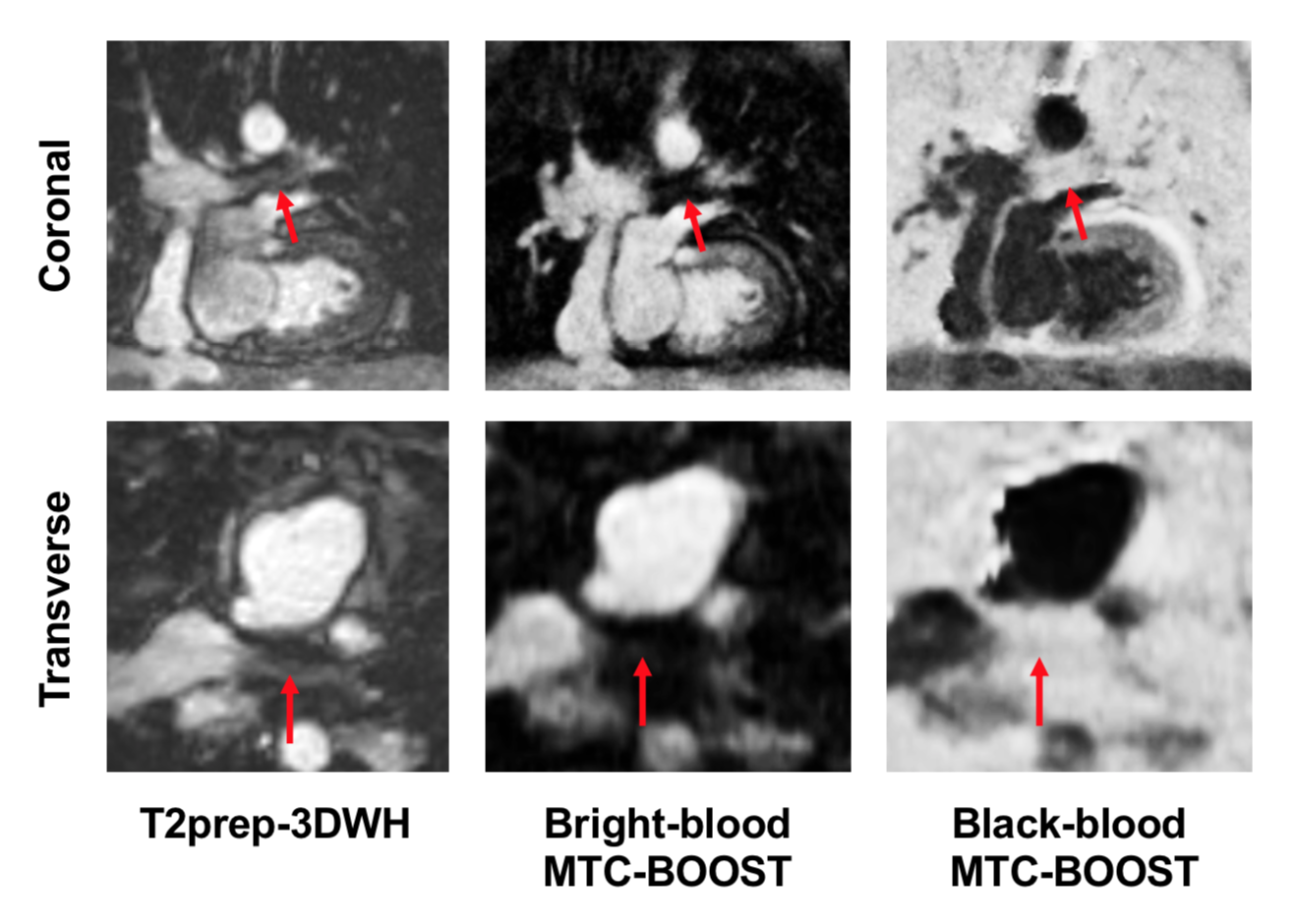
**

**Supplementary Figure 2. Bright- and black-blood MTC-BOOST and T2prep3DWH imaging of Fontan circulation with LPA stent in situ.** Coronal and transverse imaging planes are shown. The LPA stent (red arrows) is associated with luminal signal reduction/void in T2prep-3DWH, bright- and black-blood MTC-BOOST datasets.

**
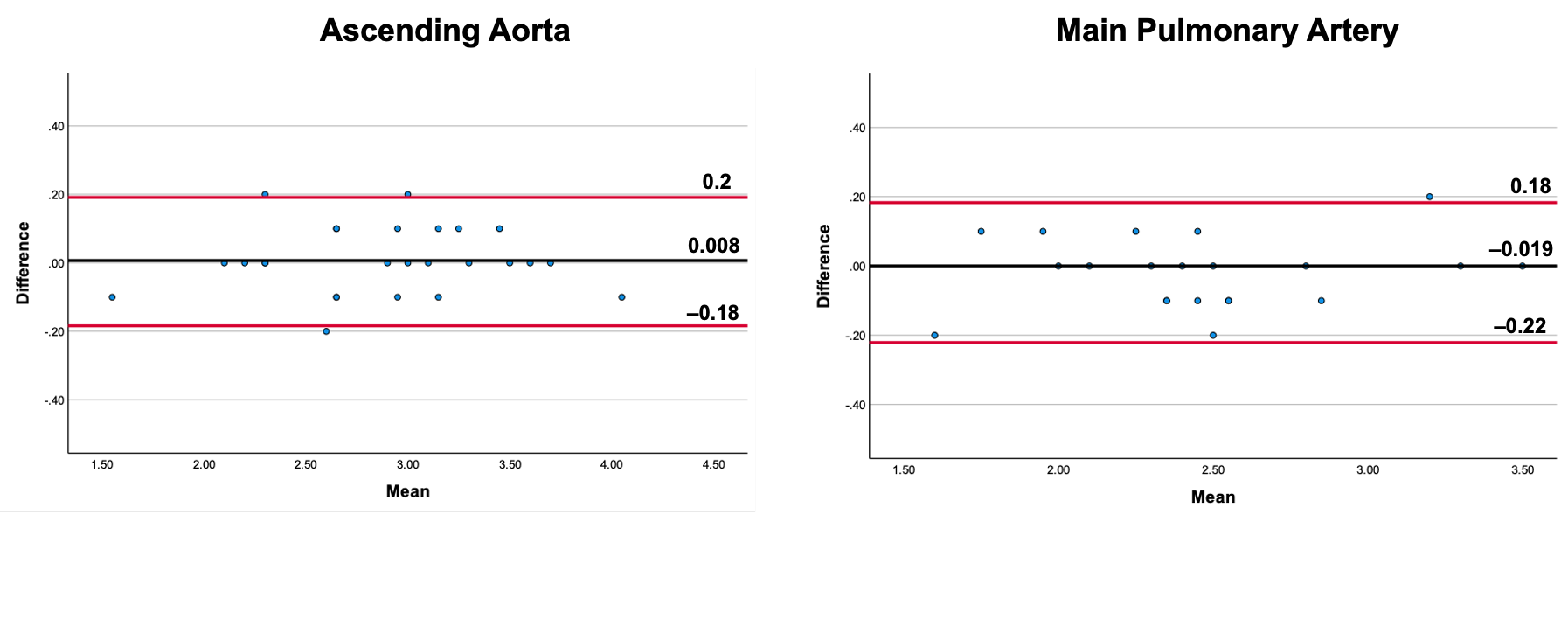
**

**Supplementary Figure 3: Bland Altman analysis of vessel dimensions derived from MTC-BOOST by different readers.** Blinded reader assessment of maximal luminal dimension (cm) of A) ascending aorta at the level of the RPA, and B) main pulmonary artery from multiplanar reformats of bright blood MTC-BOOST by two different readers (A.Fo and I.R). Black lines denote mean difference, red lines denote 95% CI (±1.96SD).

**
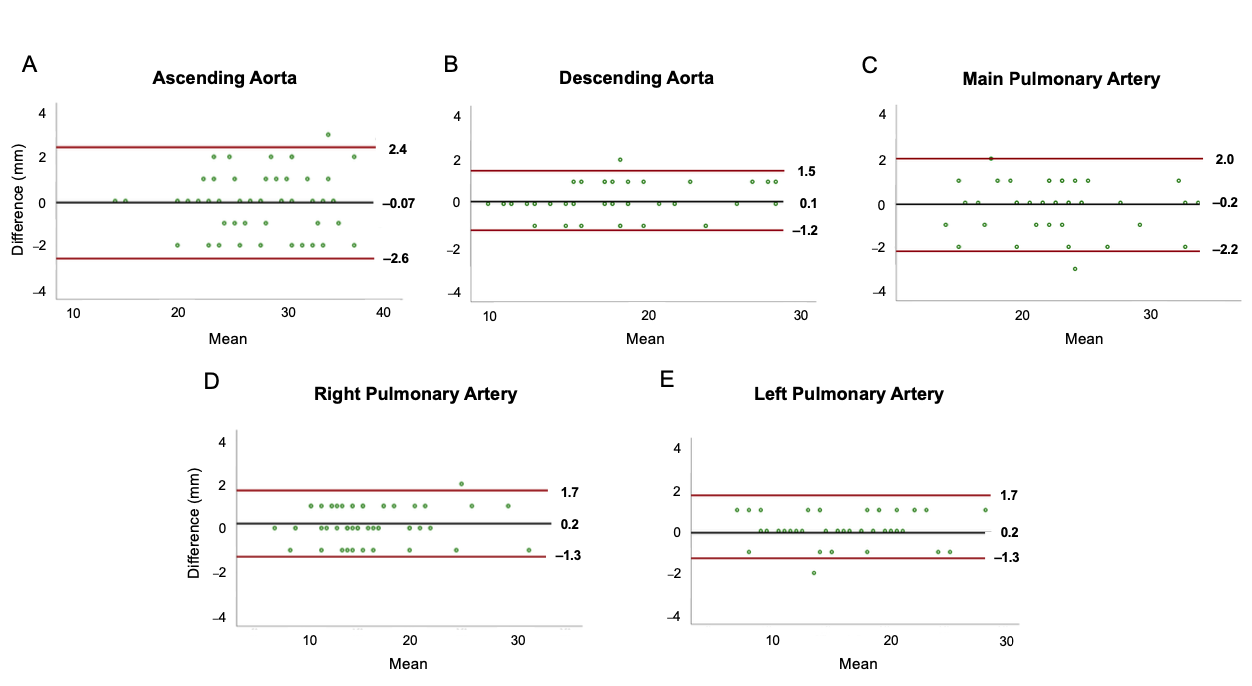
**

**Supplementary Figure 4: Bland Altman analysis of vessel dimensions using black blood MTC-BOOST and T2prep-3DWH imaging.** Blinded reader assessment of maximal luminal dimension of A) ascending aorta at the level of the RPA, B) descending thoracic aorta at the level of the RPA, C) main pulmonary artery, D) right pulmonary artery and E) left pulmonary artery from multiplanar reformats of black blood MTC-BOOST and T2prep-3DWH imaging. Black lines denote mean difference, red lines denote 95% CI (±1.96SD).


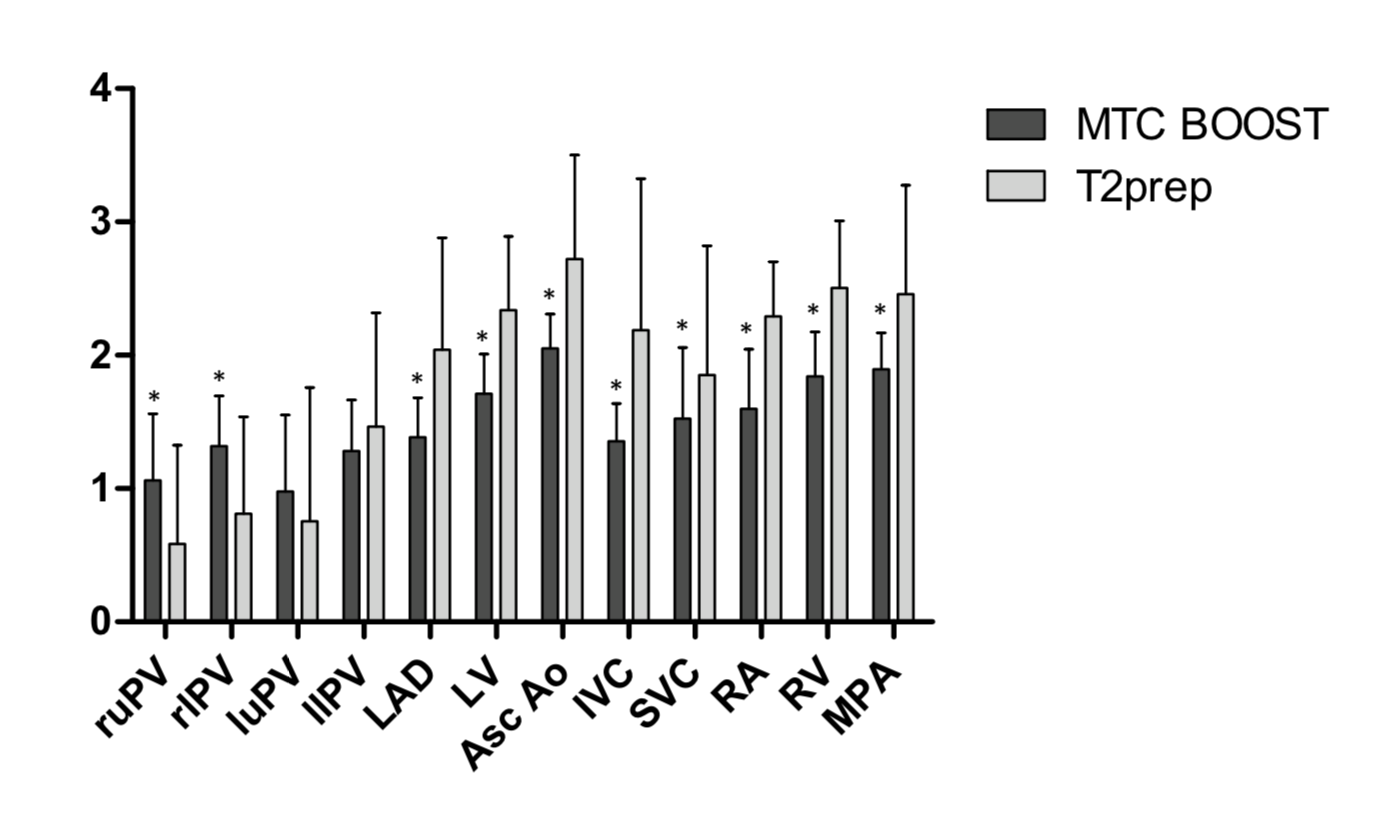


**Supplementary Figure 5: Comparison of contrast ratio (lumen/myocardium) of the great vessels and cardiac chambers using MTC-BOOST and standard clinical T2prep-3DWH imaging.** Contrast ratios of the right upper (ruPV) and right lower (rlPV) pulmonary veins were significantly higher for the right-sided pulmonary veins but not significantly different for the left upper (luPV) and left lower (llPV) pulmonary veins. Contrast ratios for the left ventricle (LV), ascending aorta (Asc Ao), inferior vena cava (IVC), superior vena cava (SVC), right atrium (RA), right ventricle (RV) and main pulmonary artery (MPA) were significantly higher for standard T2prep-3DWH imaging compared to MTC-BOOST, where all mean contrast ratios were greater than 1. *p<0.05 by Mann-Whitney.
